# Efficacy of Denture Cleansers on Microbial Adherence and Surface Topography of Conventional and CAD/CAM-Processed Denture Base Resins

**DOI:** 10.3390/polym15020460

**Published:** 2023-01-15

**Authors:** Afnan F. Alfouzan, Malath Tuwaym, Ebtihal N. Aldaghri, Tagreed Alojaymi, Hadeel Minife Alotiabi, Sara M. Al Taweel, Hanan N. Al-Otaibi, Rizwan Ali, Huda Alshehri, Nawaf Labban

**Affiliations:** 1 Department of Prosthetic Dental Sciences, College of Dentistry, King Saud University, Riyadh 11545, Saudi Arabia; 2 College of Dentistry, King Saud University, P.O. Box 60169, Riyadh 11545, Saudi Arabia; 3 Department of Prosthetic Dental Sciences, College of Dentistry, King Saud Bin Abdulaziz University for Health Sciences, Riyadh 14611, Saudi Arabia; 4 Medical Research Core Facility and Platforms, King Abdullah International Medical Research Center, King Saud Bin Abdulaziz University for Health Sciences, Ministry of National Guard Health Affairs, Riyadh 14611, Saudi Arabia

**Keywords:** 3D printing, CAD/CAM technologies, polymethylmethacrylate, acrylic resins, denture cleansers

## Abstract

This study assessed the efficacy of five denture cleansers on the microbial adherence and surface topography of conventional and CAD/CAM denture base resins. Acrylic resin discs were fabricated using conventional, milling, and 3D printing methods (N = 180). The discs were contaminated with dual species of *Candida albicans* and *Streptococcus mutans* biofilm for 72 h and then disinfected with either of the denture cleansers (Fittydent cleansing tablets, 2% Chlorhexidine gluconate, 0.2% Chlorhexidine gluconate, 0.5% sodium hypochlorite, and 1% sodium hypochlorite (n = 10). Distilled water served as the control group. The colony-forming units of the microorganisms were calculated, followed by post-treatment surface roughness. Data were statistically analyzed using one-way ANOVA, paired *t*-test, and post hoc Tukey HSD test (α = 0.05). Among the denture cleansers, 2% Chlorhexidine gluconate, 0.5% sodium hypochlorite, and 1% sodium hypochlorite had the best cleansing effect on the resin discs and demonstrated zero growth of colonies for both the species. Comparing the material groups, the 3D-processed discs showed higher colony-forming units followed by the conventional and CAD/CAM milled group. The highest surface roughness was demonstrated by the 3D-printed discs (0.690 ± 0.08 μm), followed by the conventional (0.493 ± 0.11 μm) and the milled groups (0.301 ± 0.08 μm). The tested chemical denture cleansers affected the *Candida albicans* and *Streptococcus mutans* adhesion compared to control discs immersed in distilled water. The clinician may recommend to their patient to use 2% chlorhexidine gluconate for the disinfection of CAD/CAM PMMA denture base materials.

## 1. Introduction

Despite the technological advances in dentistry, a complete denture (CD) is still the primary treatment option for the rehabilitation of the edentulous dental arch [1]. Polymethyl methacrylate (PMMA) resin has been successfully used for the fabrication of CDs for many years and is still recognized as the gold standard [2]. The advantages of using the conventional PMMA resins are the ease of handling and manipulation, non-toxicity, acceptable aesthetics, stability, and low cost [1,2,3]. On the contrary, fracture susceptibility, dimensional instability, residual monomers, surface voids, and greater risk of denture-related stomatitis limit the use of conventional PMMA resins [1,3].

In recent years, the application of computer-assisted design and computer-assisted manufacturing (CAD/CAM) technology for the fabrication of CDs has significantly expanded [2,3,4,5,6,7,8,9,10,11]. The primary CAD/CAM techniques used to fabricate CDs are the subtractive (milling) and additive (3D printing) procedures. The 3D-printed CDs use light polymers and are heat-polymerized, while the CAD/CAM-milled dentures are made from pre-polymerized PMMA discs [1,5,12]. The paramountcy of CAD/CAM-processed CDs over the conventional flask–pack–press method can be credited to enhanced surface details [13], less porosity, accuracy, reduced number of patient visits, cost-effectiveness, and quick processing time [4,10,14].

Denture stomatitis, a multifactorial oral pathological condition, is induced by biofilm that accumulates on the tissues and denture surface and is marked by varying degrees of erythema, bleeding, and halitosis [11,15]. The main causative factors in the etiology of denture stomatitis are poor oral hygiene, trauma, and *Candida albicans* infections [16,17]. However, it has been reported that multispecies biofilms, comprising *Streptococcus mutans* and *Staphylococcus aureus*, can also cause denture stomatitis rather than *C. albicans* alone [18].

Denture hygiene maintenance involves either mechanical or chemical, or a combination of both, methods [19,20]. Mechanical cleaning by brushing is a simple, easy, and cost-effective method for reducing denture biofilm; however, improper brushing can cause wear of the denture base material, leading to surface defects on the surface of the denture, which promotes bacterial colonization and pigmentation [19,21]. Furthermore, patients with low manual dexterity, especially geriatric and physically compromised patients, may find it difficult to practice the mechanical hygiene method [22]. On the contrary, the chemical method involves soaking dentures in denture cleansers for a definite amount of time, which can effectively remove food debris, biofilm, and stains from denture surfaces [21].

An ideal chemical denture cleanser should inhibit or reduce the accumulation of biofilms; possess antifungal and antibacterial activity; have a pleasant odor; and be inexpensive, easy to use, and non-toxic [17,21,23,24,25,26,27]. The importance of these treatments being efficient against microorganisms while also having minimal negative impacts on the physical and mechanical properties of the denture cannot be overstated [19,21,28]. Over the years, varieties of disinfectants have been employed successfully to limit bacterial growth and Candida species adhesion to denture surfaces. These contain one or more active ingredients, such as chlorhexidine, sodium hypochlorite, alkaline peroxides, enzymes, and diluted acids [17,21,22].

Roughness on the surface of restorative and prosthetic materials considerably interferes with material properties, decreases their longevity [21,29], and promotes microbial colonization, which indirectly causes tissue damage [30]. Previous studies have reported changes in the surface roughness of denture-base acrylic resins treated with chemical denture cleaners [21,23,31,32,33,34]. Although the CAD/CAM methods for fabricating CDs have been in existence since the 1990s, they are still viewed as a relatively new approach due to limited scientific evidence [2]. Furthermore, the clinical evidence on the best chemical protocol for disinfecting and removing biofilm from CAD/CAM processes, especially 3D-printed complete dentures, is scarce.

Therefore, this study aimed to investigate the efficacy of five denture cleansers on the microbial adherence and surface topography of conventional, milled, and 3D-printed denture base resins. The null hypothesis stated is that the tested denture cleansers would have a similar effect on the microbial adherence and surface topography of conventional, milled, and 3D-printed denture base resins.

## 2. Materials and Methods

**Figure 1:**
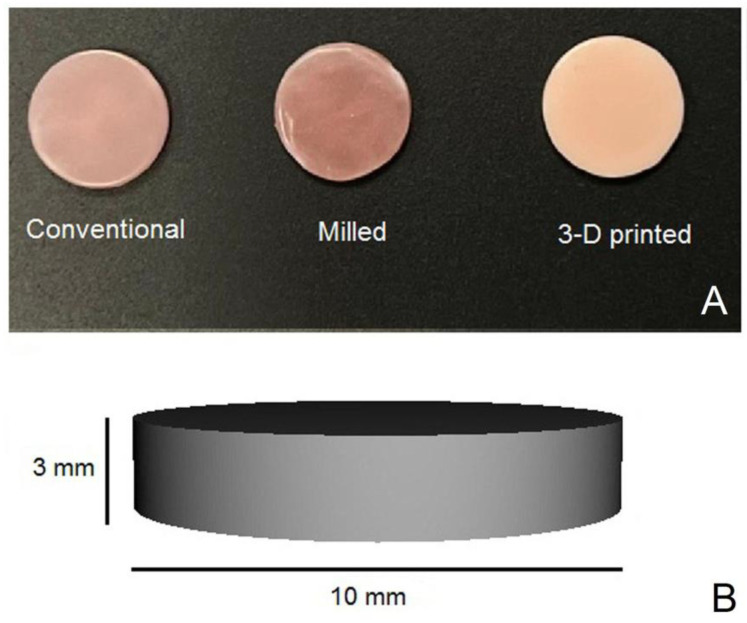
Representative disc image fabricated using three different techniques (**A**), The standard tessellation language (.STL) file of the digital disc used for fabrication of milled and 3D print discs (**B**).

PMMA acrylic resin discs (Ø10 × 3-mm) were fabricated using conventional, CAD/CAM milling, and 3D printing methods (N = 180) (Figure 1A). Table 1 lists the materials used in this study. The sample size was determined using G*Power v. 3.1.9.3 freeware (Heinrich-Heine-Universität, Düsseldorf, Germany). A minimum of 8 samples were required in each group based on an effect size of 0.45, power of 0.9, and α = 0.05.

The discs of conventional acrylic resin (Meliodent, Kulzer GmbH) were fabricated using the lost-wax technique [2]. For the fabrication of CAD/CAM-milled discs, Zintec CAD software (Ivoclar Vivadent, Schaan, Liechtenstein) was used to model the disc according to the specified dimensions digitally. The standard tessellation language (STL) file of the digital disc obtained through CAD software (Figure 1B) was imported to the Zenotec CAM software (Ivoclar Vivadent, Schaan, Liechtenstein), and a pre-polymerized CAD/CAM PMMA block (IvoBase CAD, Ivoclar Vivadent, Schaan, Liechtenstein) was milled using a 5-axis simultaneous milling machine (Zenotec^®^ selection, Ivoclar Vivadent, Schaan, Liechtenstein).

The 3D-printed discs were fabricated using the same digital file used for milling. The STL file was uploaded to the CAM software (Chitubox, Guangdong, China) connected to a Mask Stereolithography (MYLA) 3D printer (ST-1600, Satori Ltd., London, UK). The photopolymerized liquid resin (Denture 3D+, NextDent, Soesterberg, The Netherlands) was used to print the disc layer-by-layer at a 0-degree direction and at a thickness of 50 µm using the MSLA technique [1,14]. Once the 3D-printed discs were processed, the supports from the discs were removed and cleaned in an ultrasonic tank containing isopropanol. Then, the discs were subjected to post-process polymerization by immersion in glycerin for 40 min in a post-curing oven (Zirlux, Zahn Dental Labs, Henry Schein, New York, NY, USA) [35].

For standardization purposes, a single investigator performed the finishing and polishing of all the acrylic discs. The discs were finished with a tungsten carbide bur (Hager & Meisinger GmbH, Neuss, Germany) at 300 rpm under water coolant and then polished in a compact polishing unit (M2line, Manfredi, San Secondo di Pinerolo, Italy) using a wet rag wheel and pumice slurry at high speed for 60 s. The final gloss of the disc was obtained using a dry and clean rag buff wheel and zircate prophy paste (Dentsply Caulk, Milford, DE, USA). The discs were ultrasonically cleaned in distilled water for 5 min and dried with absorbent paper. Next, the discs were randomly allocated to 6 groups using the lottery method (n = 10).

The surface roughness of the acrylic resin disc was assessed using an optical non-contact profilometer (Contour GT-X, Bruker, Billerica, MA, USA) assembled with the atomic force microscopy (AFM) module and Vision 64 (v.5.3, Bruker, Billerica, MA, USA) proprietary software [36]. The measurement was performed using vertical scanning interferometry, which uses a white light source and is effective for analyzing objects with rough surfaces and pixel-height differences greater than 135 nm. The measurement parameters included a 1 × 1 mm^2^ field of view, a scan speed of 1×, and a 0.1 mm/s stage speed. The disc was scanned at 5 equidistant areas, and the mean of these readings correlates to that particular disc’s surface roughness and is expressed as Ra (μm).

Biofilm assays were performed with dual-spp biofilms of *C. albicans* and *S. mutans*. The *C. albicans* strain (ATCC 10231) and *S. mutans* strain (ATCC 25175) were harvested from a fresh culture and inoculated into 5 mL of brain heart infusion (BHI; Merck KGaA, Darmstadt, Germany) broth and incubated at 37 °C for 48 h. The aftermath cell pellets were washed twice with phosphate-buffered saline (PBS; Merck KGaA, Darmstadt, Germany) for 2 s to remove non-adherent cells. After dilution with PBS, the final dual-spp cell suspension was prepared to a concentration of 1 × 10^−^^3^ cells/mL. The discs were placed in petri dishes (SPL Life Sciences Co., Ltd., Gyeonggi-do, Republic of Korea) containing 1 mL of BHI broth and 12.5 μL of prepared cell suspension. The discs were incubated at 37 °C for 72 h at 75 rpm to permit biofilm formation (adhesion period).

Five denture cleansers—Fittydent cleansing tablets (Fittydent International GmbH, Pinkafeld, Austria), 0.2% Chlorhexidine gluconate (CHG) (Avalon Pharma, Riyadh, Saudi Arabia), 2% CHG (Prevest Denpro Limited, Jammu, India), 0.5% sodium hypochlorite (NaOCl), and 1% NaOCl (Biotischen Industry Incorporation, Alkharj, Saudi Arabia)—were prepared according to the manufacturer’s instructions. The discs were immersed in 2 mL of prepared denture cleanser solution in accordance with previous studies, and then Fittydent solution for 5 min [37], 0.2% CHG for 20 min [21], 2% CHG for 5 min [21], 0.5% NaOCl for 20 min [38], and 1% NaOCl for 10 min [24]. The discs in the control group were not subjected to any treatment but were immersed in distilled water for 20 min. After the treatment, each disc was removed from the solution and gently washed twice with PBS to remove the non-adherent cells.

The adherent microbial cells were dislodged from the surfaces of the discs in 2 mL of distilled water by vortexing for 1 min using a super mixer. The cell suspension was diluted to 4 by transferring 100 μL of the cell suspension to 900 μL of distilled water each time. The third and fourth dilutions were then plated on Sabouraud Dextrose Agar (SDA; Merck KGaA, Darmstadt, Germany) medium and incubated at 37 °C for 24 to 72 h under aerobic conditions. After 72 h of incubation, the number of colonies formed was counted for each dilution and expressed as a colony-forming unit (CFU)/mL [39].

The cell viability of the microorganism was assessed using Eclipse TE2000 confocal laser scanning microscope (CLSM) (Nikon Corporation, Tokyo, Japan). The discs with adherent biofilm were washed thrice with PBS and stained with Cell Tracker™ green, actin red, and Hoechst 33342 blue dyes (Thermo Fisher Scientific, Waltham, MA, USA) for 1 h. Post-staining images of the discs were acquired using an Argon laser (Eclipse TE2000 confocal laser scanning microscope (CLSM), Melville, NY, USA) at 488/520–530 nm (ex/em) for Cell Tracker™ Green and a HeNe laser at 633/650 nm (ex/em) for actin red. The counterstained nucleus was detected using a UV laser at 350/460 nm (ex/em). The specimens were observed using a 4× lens, and images were acquired using an oil immersion objective. Five random stacks spanning the entire surface of the disc with a step size of 2 µm were acquired for each representative specimen.

A representative disc from each group was subjected to qualitative analysis using a scanning electron microscope (Jeol JSM-6610LV, Tokyo, Japan) to determine the pattern of microbial adhesion or growth on the surface. The representative disc after SEM analysis was not counted for further microbiological analysis. Before image processing, the disc was gold sputter-coated for 1 min using a coating machine (Quorum Q150R, Essex, MA, USA). The SEM images were acquired at a magnification of 5000×, 15 kV, and a working distance of 10 mm. In both CSLM and SEM analysis, the images of a contaminated denture surface of the negative control group were used as a reference for comparison.

The data analysis was performed using Statistical Package for Social Sciences (SPSS) (v.20, IBM SPSS Inc., Armonk, NY, USA). One-way ANOVA was used to describe the biofilm isolate adhesion values (CFU) and surface roughness of the conventional, milled, and 3D-printed discs. A paired t-test was used to compare the surface roughness of each material group before and after denture cleanser treatment. A post hoc Tukey HSD test was used to find any significant interactions between the material groups (α = 0.05).

## 3. Results

Table 2 presents the mean and SD number (10^3^ CFU/mL) of C. albicans and *S. mutans* after denture cleanser treatment. Among the denture cleansers evaluated, 2% CHG, 0.5% NaOCl, and 1% NaOCl had the best cleansing effect on the resin discs. The results of the CFU/mL for the discs treated with the above denture cleanser solution were zero for both the spp.

The discs treated with Fittydent solution showed high CFU/mL for *C. albicans* spp, and the difference in the number of CFU/mL between the different material groups was statistically significant (*p* = 0.003). For the *S. mutans* spp, the discs immersed in water showed high CFU/*mL* compared to the discs immersed in cleansing solutions; however, there was no significant difference in CFU/mL between the material groups (*p* = 0.29). The CFU/mL of the discs treated with Fittydent solution showed a statistically significant difference between the material groups (*p* = 0.017). In comparison to the Fittydent group, the discs treated with 0.2% CHG demonstrated higher CFU/mL for the conventional and milled group and lower CFU/mL for the 3D-printed discs.

Comparing the CFU/mL of the material groups, the 3D-processed discs showed higher values followed by the conventional group. The CAD/CAM-milled discs demonstrated the lowest CFU/mL of all the material groups.

**Figure 2:**
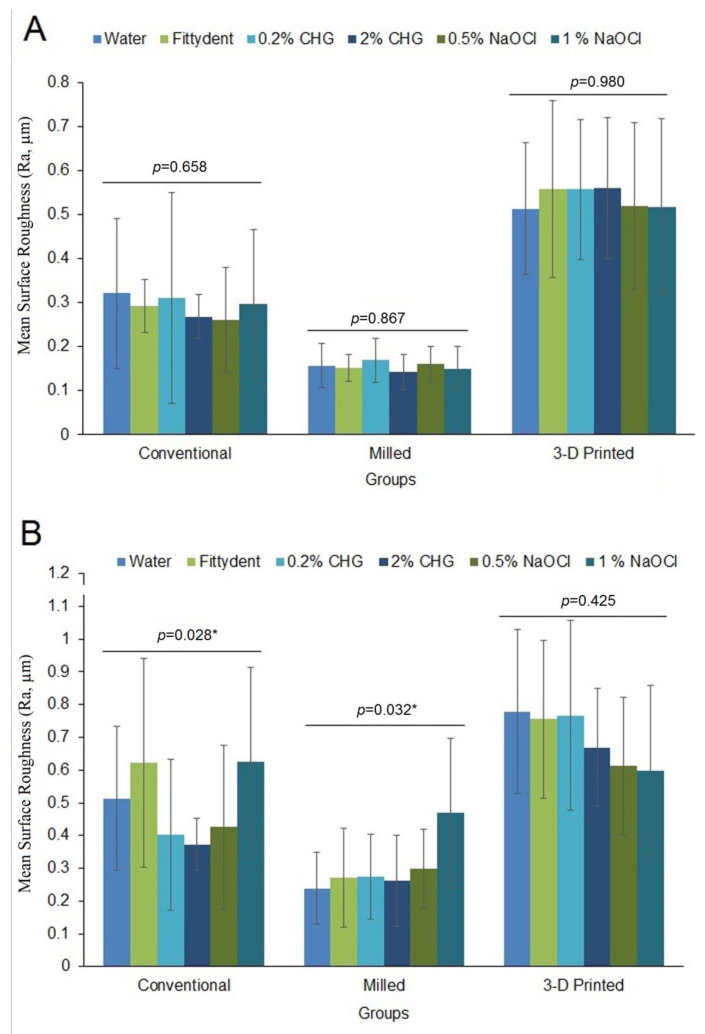
Mean Surface Roughness (Ra) of the material groups. (**A**) Pre-treatment, (**B**) Post-treatment either in distilled water or denture cleanser solutions. Bars indicate SD. * indicate significant difference within the materials (conventional and milled) after surface treatment with denture cleansers.

Figure 2 presents the mean pre- and post-treatment Ra of the material groups. The lowest pre-treatment Ra was demonstrated by the milled group (0.155 ± 0.04 μm), followed by the conventional (0.291 ± 0.13 μm) and 3D-printed groups (0.537 ± 0.17 μm). After denture cleanser treatment, all the material groups demonstrated significantly increased Ra values compared to pre-treatment values (*p* < 0.05). The highest Ra was demonstrated by the 3D-printed discs (0.690 ± 0.08 μm), followed by the conventional (0.493 ± 0.11 μm) and the milled groups (0.301 ± 0.08 μm). The comparison between the surface-treated discs of the conventional and milled groups showed a significant difference in Ra for the denture cleansers used (*p* = 0.028 and *p* = 0.032, respectively).

**Figure 3:**
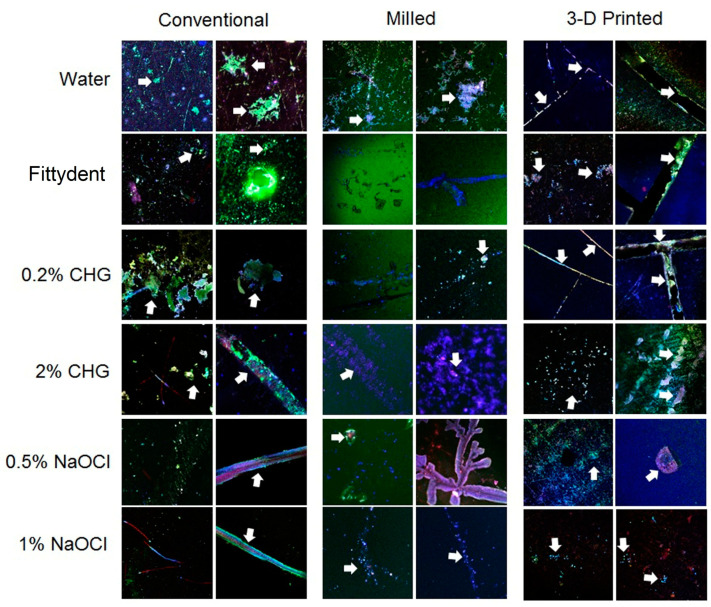
Confocal laser scanning microscopy (CLSM) images of the post-treatment discs from each material group. The sample discs were stained with HOECHST33342 (blue), Cell Tracker™ Green (green), and Actin Red (red). White arrow shows adherent microbes on the discs. Conventional and 3D-printed discs showed most of the microbe growth as evident in the images. Overall, Sodium-Hypochlorite-treated samples showed lowest microbe growth in comparison to other groups.

Figure 3 presents the CSLM images of the representative discs from each material group treated with denture cleansers. Among the groups, 3D-printed samples demonstrated increased microbial adherence, especially in the grooves compared to the conventional and CAD/CAM-milled groups. The NaOCl-treated samples showed the lowest microbial adherence in comparison to other denture cleansers.

**Figure 4:**
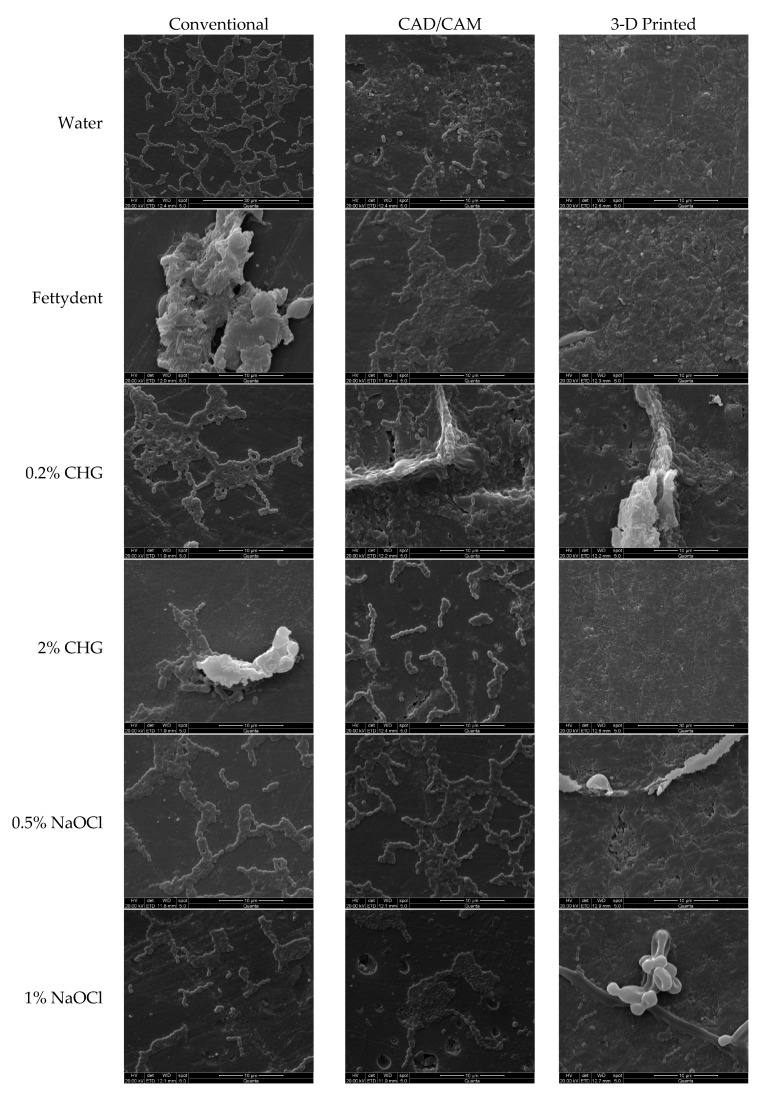
Scanning electron microscopy (SEM) images of the representative disc demonstrating the microbial growth on the surface.

The SEM micrographs demonstrating the microbial adherence to the disc surface for all the groups are presented in Figure 4. A larger area of the disc was covered with densely packed microorganisms on the 3D-printed dentures, followed by less colonization and loosely packed microorganisms on the conventional and CAD/CAM-milled surfaces.

## 4. Discussion

To the best of the authors’ knowledge, there are no previous studies which evaluated the efficacy of denture cleansers on the microbial adherence and surface topography of CAD/CAM-milled and 3D-printed PMMA resins. The null hypothesis was partially rejected in light of the study outcomes, as the tested denture cleansers did not show a similar effect on the microbial adherence and surface topography of the tested denture base resins.

Denture stomatitis is a common problem affecting 15–70% of denture wearers [40]. In the current study, the dual-spp strains of *C. albicans* and *S. mutans* were employed based on the pathogenic potential or representational significance of the microorganisms for antimicrobial-effectiveness evaluation studies. *C. albicans* is associated with the occurrence of denture stomatitis in conjunction with other factors such as denture hygiene, trauma, and systemic diseases. Therefore, research in dentistry focuses on *C. albicans* as a role in stomatitis as well as a concern with cross-infection. *S. mutans* is a normal component of the oral microbiota; however, the presence of this microbe at the infection site could be interpreted as a contamination indicator [23].

A short-term chemical disinfection protocol helps treat denture stomatitis. Among the several chemical denture cleansers available on the market, NaOCl and CHG chemical solutions are widely used [41], although the concentration and exposure time for effective disinfection are still conflicting [39]. Sodium hypochlorite exhibits a wide range of activity with a shorter duration and is inexpensive. However, as a disinfectant, NaOCl is corrosive and irritating to skin and mucous membranes [23]. Previous studies have demonstrated the exceptional disinfection activity of 0.5% [39] and 1% [42] NaOCl in inhibiting *C. albicans* and *S. mutans* from the denture surface. On the contrary, CHG is considered the best chemical denture cleanser for controlling dental biofilm and preventing denture stomatitis. It can bind to oral surfaces with a gradual release, effectively limiting the initial adherence of fungus and other microbes and, as a result, minimizing biofilm formation [43]. Chlorhexidine gluconate has been successfully used as an antiseptic mouthwash in the treatment of denture stomatitis at 0.2% concentration, while at 2.0% concentration, it is used as an overnight denture disinfection solution [21].

The outcome of this study demonstrated that short-term chemical disinfection protocols using 0.5% and 1% NaOCl and 2% CHG effectively reduced the *C. albicans* and *S. mutans* cell adhesion. The CFU/mL of the PMMA discs (irrespective of the processing method) treated with these solutions was low for both the spp, indicating the best denture-cleansing effect, and thus agreeing with previous studies [23,29,39,42], with a similar outcome. In contrast, CHG at a lower concentration (0.2%) was ineffective in completely removing *C. albicans* from 3D-printed discs and *S. mutans* from either of the disc surfaces. This outcome is in disagreement with the findings of de Andrade et al. [21] where the authors concluded that immersion in 0.12% CHG had a similar ability as 2% CHG to remove denture biofilms. The results of this study also demonstrated that despite being recommended as prosthetics cleaners, the commercial Fittydent tabs did not exhibit adequate antimicrobial activity. In contrast, Kim et al. [44] showed a strong plaque-removing effect and bacteriostatic effect with the use of Fittydent tabs. However, the authors of the current study care to mention that a comparison with previous studies should be performed with caution due to the differences in methodological approach, materials, and denture cleansers tested.

Comparing the CFU/mL of the discs processed using different techniques, the 3D-processed discs showed higher values followed by the conventional group. The milled discs demonstrated a lower CFU/mL of all the material groups. This outcome supports the predominance of CAD/CAM milling over 3D printing, as also concluded by previous studies [2,12]. On the contrary, Schubert et al. [11] found that *S. mutans* adhesion was unaffected, whereas *C. albicans* adhesion increased on milled and 3D-printed oral splints compared to traditional thermoforming and pressing. Similarly, after 16 h of incubation, Fiore et al. [45] found no noticeable difference between the average microbial adhesion values of milled, 3D-printed, and conventional PMMA resins. However, it is worth mentioning that no disinfection protocol was followed in the previous studies.

Surface roughness plays a significant role in microbial colonization on denture surfaces [17,46]. The irregularities or imperfections on the denture surface enhance the microbial accumulation even on a clean prosthesis [47]. A roughness threshold of 0.2 µm has been recommended to prevent biofilm formation on the dental hard and prosthetic surfaces [45]. To provide comprehensive knowledge about the disc surface following treatment, the quantitative roughness measurements were complemented with qualitative scanning electron microscopy and fluorescence laser scanning microscopy (LSM) analysis in the current treatment.

Despite a similar finishing and polishing by a single operator, the PMMA material groups exhibited roughness values above the threshold values except for the milled PMMA discs at baseline. After treatment, all the PMMA material groups showed a significant increase in roughness. However, the milled discs exhibited Ra values below the threshold limit except for the samples immersed in 1% NaOCl. The specimens stored in 2% CHG showed the lowest roughness values among the denture cleansers used except for the 3D-printed group, where it showed values non-significantly higher than discs immersed in NaOCl. Although previous studies have compared the surface roughness between the three processing methods [11,45], the effect of denture cleansers on denture disinfection was not evaluated. Thus, there was no relevant comparison of the current results with the earlier data.

Although the purpose of the current in vitro investigation was to mimic a natural intraoral environment, it has some limitations. The standard methodological approach may not be logical in in vivo conditions. The antimicrobial property of saliva could contribute to the decreased adhesion of microorganisms, which was not considered in the present study. Depending on the type of denture-cleanser solution, the immersion period varies. However, for individuals who practice good denture hygiene frequently, the actual immersion time may differ. Future studies should focus on evaluating the role of an acquired salivary pellicle in microbial adhesion and the efficacy of disinfection protocols of the tested CAD/CAM materials. In addition, the tested parameters should be evaluated in an in vivo model to clearly understand the behavior of these materials and denture cleansers in an actual clinical environment.

## 5. Conclusions

Considering the methodological approach and the limitations, the tested chemical denture-cleanser solutions affected *Candida albicans* biofilm and *Streptococcus mutans* adhesion; however, all denture cleansers significantly increased the surface roughness of the PMMA discs. Irrespective of the processing method, the results showed a high antimicrobial effect of 2% CHG, 0.5%, and 1% NaOCl cleansers. However, considering the roughness parameter, 2% CHG was a more promising denture cleanser for the disinfection of CAD/CAM-processed dentures. The clinician may recommend to their patient to use 2% chlorhexidine gluconate for the disinfection of CAD/CAM PMMA denture base materials.

## Figures and Tables

**Table 1:** Materials used in the study.

Material	Lot No.	Composition	Manufacturer
IvoBase CAD	UP0897	Industrially polymerized blocks containing >90% polymethylmethacrylate	Wieland Digital Denture (Danbury, CT, USA)
Denture 3D+	WY032N01	90% methacrylic oligomers, methacrylate monomer, phosphine oxides, and pigment	NextDent (Soesterberg, The Netherlands)
Meliodent heat cure acrylic resin	K010028	Powder: Polymethylmethacrylate, ethyl hexyl acrylate, N-octyl methacrylate Liquid: methyl methacrylate, glycol dimethacrylate, dimethyl p-touludine	Heraeus Kulzer GmbH (Hanau, Germany)

**Table 2:** Number (10^3^ CFU/mL) of *C. albicans* and *S. mutans* after denture cleanser treatment (Mean ± SD).

	Denture Cleansers	Conventional	CAD/CAM	3D-Printed	*p* Value
		Mean ± SD	Mean ± SD	Mean ± SD	
*C. albicans*	Water	14.3 ± 13.1	7.7 ±5.8	5.0 ± 5.8	0.20
	Fittydent	17.2 ± 13.6	2.2 ±1.6 ^a^	32.1 ± 22.6 ^b^	0.003 *
	0.2% CHG	0.00 ± 0.00	0.00 ± 0.00	4 ± 4.2	n/a ^†^
	2% CHG	0.00 ± 0.00	0.00 ± 0.00	0.00 ± 0.00	n/a ^†^
	0.5% NaOCl	0.00 ± 0.00	0.00 ± 0.00	0.00 ± 0.00	n/a ^†^
	1% NaOCl	0.00 ± 0.00	0.00 ± 0.00	0.00 ± 0.00	n/a ^†^
*S. mutans*	Water	288.4 ± 64.4	327.9 ± 47	245.4 ± 577.7	0.29
	Fittydent	35.7 ± 25.6	16.3 ± 19.5	92.3 ± 126.2 ^a^	0.017 *
	0.2% CHG	79.1 ± 101.3	16.5 ± 12	44.8 ± 49.7	0.74
	2% CHG	0.00 ± 0.00	0.00 ± 0.00	0.00 ± 0.00	n/a ^†^
	0.5% NaOCl	0.00 ± 0.00	0.00 ± 0.00	0.00 ± 0.00	n/a ^†^
	1% NaOCl	0.00 ± 0.00	0.00 ± 0.00	0.00 ± 0.00	n/a ^†^

* statistically significant between the groups (*p* < 0.05). Superscript a and b alphabets represent post hoc analysis significance for conventional and CAD/CAM, respectively, for *C. albicans* and significance between them. ^†^ Test not performed, because the discs in these groups had 0 CFU.

## Data Availability

Data sharing is not applicable to this article.
